# COVID-19 - Computed tomography findings in two patients in Petrópolis, Rio de Janeiro, Brazil

**DOI:** 10.1590/0037-8682-0147-2020

**Published:** 2020-04-22

**Authors:** Bernardo Carvalho Muniz, Miguel Angelo Milito, Edson Marchiori

**Affiliations:** 1 Hospital Santa Teresa, Departamento de Radiologia, Central Integrada de Imagens - LUMIC, Petrópolis, RJ, Brasil.; 2 Universidade Federal do Rio de Janeiro, Departamento de Radiologia, Rio de Janeiro, RJ, Brasil.

A 65-year-old male patient (patient 1) previously healthy, returned from a trip to Egypt with a stopover in New York in March 2020. After 5 days, he presented coryza, asthenia, myalgia, dry cough, unverified fever and mild dyspnea. His 67-year-old wife (patient 2), who also accompanied him on the trip, developed the same symptoms the next day. Two days after the onset of patient 1 symptoms, the condition worsened and the patients went to the Emergency Room. Upon examination, he was afebrile, with severe dyspnea, respiratory rate of 20 breaths per minute and oxygen saturation of 90%, normotensive, acyanotic. She was afebrile, eupneic, with 95% oxygen saturation, normotensive, acyanotic. Both patients did not present significant changes in laboratory tests.

**FIGURE 1:**
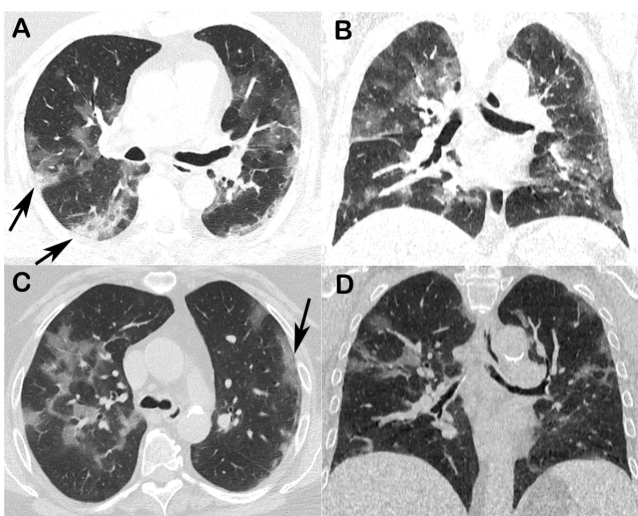
A - Patient 1, chest CT, pulmonary window, axial (A) and coronal (B) reconstructions showing multifocal areas of ground-glass opacities in both lungs, with small areas of consolidation in the right lower lobe (arrow). C and D (patient 2), chest CT with pulmonary window, showing multifocal areas of ground-glass opacities in both lungs. Note subpleural and peripheral involvement in the lingula (arrow).

Chest radiographs were normal. Chest CT showed bilateral multifocal ground-glass opacities in both patients, some associated with areas of consolidation (Figure 1). There was no pleural effusion or lymph node enlargement.

Patients were considered suspicious for COVID-19, and collection of material to conduct the SARS-CoV-2 research by polymerase chain reaction (PCR), was performed. The patients were admitted to the intensive care unit, in isolation. Positive PCR result confirmed the diagnosis of COVID-19 in both patients. Patient 1 evolved with respiratory failure, renal failure, and death. Patient 2 remained stable, with clinical improvement, still hospitalized, no longer in the intensive care unit.

Radiological examinations are of great importance in the early detection and treatment of COVID-19. Chest radiography is not sensitive for the early detection of the disease and may demonstrate normal findings in the initial stage of infection^1^ ^,^ ^2^, and is not recommended as the first-line imaging modality for COVID-19. 

High-resolution chest CT is the most effective radiological examination for the early detection of lung involvement by COVID-19. The largest sample study to date has shown that, among 3,665 confirmed cases, in 95.5% (n = 3,498) of the patients, pulmonary impairment was correctly diagnosed by CT^1^, which provides valuable information for diagnosis and evaluates the severity of lung disease caused by COVID-19, guiding clinical treatment.
